# Lentils pasta by-product in a complete extruded diet for dogs and its effect on extrusion, digestibility, and carbohydrate metabolism

**DOI:** 10.3389/fvets.2024.1429218

**Published:** 2024-06-27

**Authors:** Livio Penazzi, Ticiane Giselle Bitencourt Freire, Stephanie de Souza Theodoro, Juliana Lopes Frias, Ugo Ala, Aulus Cavalieri Carciofi, Liviana Prola

**Affiliations:** ^1^Department of Veterinary Sciences, University of Turin, Grugliasco, Italy; ^2^Department of Veterinary Clinic and Surgery, Universidade Estadual Paulista (UNESP), Jaboticabal, São Paulo, Brazil

**Keywords:** canine, sustainability, pet food, by-products, nutrition, processing, digestibility, palatability

## Abstract

**Introduction:**

Recently, increasing effort has been directed toward environmental sustainability in pet food. The aim of this study was to evaluate the extrusion parameters, nutrient digestibility, fecal characteristics, palatability and insulinemic and glycaemic curves of a complete diet for dogs in which the main carbohydrate source was a red lentil pasta by-product (LP).

**Methods:**

Five experimental diets were formulated: a basal diet (CO) based on rice and a poultry by-product meal; three experimental diets where LP substituted rice at 33, 66, or 100% (LP33, LP66, and LP100, respectively); and a diet formulated on 70% of the basal diet (CO) plus 30% LP (LPS) to evaluate the digestibility of LP ingredient.

**Results and discussion:**

The extruder pressure, hardness and bulk density of the kibble increased in a linear manner with increasing LP percentage (*P* < 0.05), without affecting starch gelatinization. According to polynomial contrast analysis, rice replacement with LP at 33 and 66% caused no reduction in apparent total tract digestibility coefficient (ATTDC), with similar or higher values compared with the CO diet. Nitrogen balance did not change (P > 0.05), but we observed a linear increase in feces production and moisture content as the LP inclusion rate rose and a linear decrease in feces pH (*P* < 0.05). Nevertheless, the fecal score was unaffected. Fecal acetate, propionate, total short-chain fatty acids (SCFA), branched-chain fatty acids, and lactate all increased linearly as the LP inclusion rate increased (*P* < 0.05), without altering ammonia concentration in feces. Feces concentrations of cadaverine, tyramine, histamine, and spermidine also increased in a linear manner with increasing LP inclusion (*P* < 0.05). The fermentation of LP dietary fiber by the gut microbiota increased the concentration of desirable fermentation by-products, including SCFA and spermidine. The post-prandial glucose and insulin responses were lower in the dogs fed the LP100 diet compared with CO, suggesting the possible use of this ingredient in diets designed to generate a low glycaemic response. Finally, the palatability study results showed a preference for the LP100 ration in both the “first choice” and the “consumption rate” evaluation (*P* < 0.05). This trial reveals how a by-product discarded from the human-grade food chain retains both its nutritional and organoleptic properties.

## 1 Introduction

The production of protein sources demands substantial land and water usage, making them food ingredients with a high ecological impact, in addition to being expensive (1). Nevertheless, protein (as a macronutrient) is of key importance for pet owners when choosing food for their pets (2–4). The use of alternative raw materials, such as by-products from the food industry, offers a way of improving nutrient provision and pet food sustainability in terms of both economics and the environment (5, 6). With the livestock industry at its limit in terms of sustainable production capacity, and the pet food business in constant growth, new sources of protein are being sought to meet the market's demand and the expectations of pet owners (7, 8). Thus, the revalorization of food by-products, otherwise destined to compost or landfill, should be prioritized. In particular, although the concept of upcycling, i.e., using still nutritionally valuable but discarded by-products, is already well established in the pet food industry, it is presently the focus of renewed research interest in various fields, including marketing, considering the magnitude of the recent food waste crisis (9–11). From an environmental standpoint, plant-based diets require less energy, land and water usage compared with meat-based diets (1, 12). Plant proteins can overcome their nutritional deficiencies if combined with the proper meat-based ingredients and easily form part of the canine diet (13). To favor the use of more sustainable ingredients in our pets' diets, scientific investigations are needed to ensure animal food safety (14). Legumes have been rediscovered by the pet food industry alternative sources of carbohydrate and protein, and they are particularly interesting for the fast-growing “grain-free” market (15). Although starch is not considered an essential nutrient for dogs, it forms a major source of energy in pet food formulations and is essential for the extrusion process and kibble formation (16–22). Different factors may affect starch digestibility and animal metabolism, such as the carbohydrate source (23–27), starch type (28, 29), processing conditions (27, 30, 31), starch-protein interactions (31, 32) and physical granule form (29, 32). These factors have been thoroughly addressed in dog foods based on traditional cereals, but alternative carbohydrate sources have received comparatively little attention, especially pulses (17, 33). The production of lentil pasta is steadily growing due to the growing incidence of gluten intolerance and celiac disease (34). As a result, the availability of lentil pasta by-product (discarded due to discoloration or not being perfectly shaped) is also growing and attracting the attention of the pet food industry as a possible alternative ingredient for dogs. Lentil-based products may be useful for specific dietary conditions such as diabetes mellitus, obesity, pregnancy, stress, infection, cancer and senescence, as it minimizes (and prolongs) the postprandial glucose and insulin responses, possibly improving glycaemic control (24). The aim of this study was to assess the extrusion parameters, digestibility, acceptance and metabolic responses of dogs fed an extruded complete diet containing a red lentil pasta by-product.

## 2 Materials and methods

### 2.1 Diets and digestibility protocols

#### 2.1.1 Experimental design and extrusion study

The study was performed at the Universidade Estadual Paulista (UNESP), Faculdade de Ciências Agrárias e Veterinárias, Jaboticabal (São Paulo), Brazil. All the experimental procedures were approved by the Bioethics Committee (CEUA) of the “Universidade Estadual Paulista” (UNESP) –Jaboticabal Campus (Brazil) (prot. n. 1501/21).

Forty adult, clinically healthy, beagle dogs [19 males and 21 females, all neutered, median age 3 years (interquartile range (IQR) 2–7), 11.9 ± 2.0 kg BW, with a median BCS of 5 (on a 9-point scale, IQR 5–6) (35)] were randomly divided into five groups. Each group received a different dry extruded diet, formulated to supply the nutritional requirements of adult dogs according to FEDIAF (36). One group received the basal diet (CO) based on rice and a poultry by-product meal. Three groups each received an experimental diet in which 33, 66 or 100% of the rice was substituted with a red lentil pasta by-product (LP) (LP33, LP66 and LP100, respectively). As the other ingredients were not altered in the formula, the final chemical composition varied between groups. The fifth experimental diet (LPS) was formulated to contain 70% of the basal diet (CO) plus 30% of the lentil pasta by-product. This diet was used to evaluate LP digestibility. The ingredient composition and particle size of each diet is shown in Table 1.

**Table 1:** Ingredients composition and particle size of the experimental diets.

***Ingredients* (g/kg, as-fed basis)**	**Experimental diets** ^*****a*****^			
	***CO***	***LP33***	***LP66***	***LP100***
Broken rice	571.7	381.2	190.6	-
Red lentil pasta by-product	-	190.6	381.2	571.7
Poultry by-product meal	281.8	281.8	281.8	281.8
Poultry fat	86.4	86.4	86.4	86.4
Palatability enhancer^*b*^	20.0	20.0	20.0	20.0
Beet pulp	20.0	20.0	20.0	20.0
Potassium chloride	4.7	4.7	4.7	4.7
Sodium chloride	4.5	4.5	4.5	4.5
Vitamin-mineral premix^*c*^	3.2	3.2	3.2	3.2
Mold inhibitor^*d*^	1.0	1.0	1.0	1.0
Antioxidant^*e*^	0.5	0.5	0.5	0.5
DL-Methionine	0.2	0.2	0.2	0.2
Mean geometric diameter (μm)^*f*^	208	223	211	224
Geometric standard deviation (μm)	1.45	1.28	1.38	1.28

^*a*^CO, control diet; LP33, substitution 33% rice with red lentil pasta by-product; LP66, substitution 66% rice with red lentil pasta by-product; LP100, substitution 100% rice with red lentil pasta by-product. ^*b*^D'TECH 10L, Palatabilizante Líquido, SPF do Brasil Indústria e Comércio Ltda., Descalvado, Brazil. ^*c*^Added per kg of food: Vitamin A, 24, 000 IU; Vitamin D3, 1, 350 IU; Vitamin E, 150 IU; Vitamin K3, 1.5 mg; Vitamin B1, 4 mg; Vitamin B2, 9 mg; Pantothenic Acid, 20 mg; Vitamin B6, 3 mg; Vitamin B12, 54 mcg; Vitamin C, 90 mg; Nicotinic Acid, 25 mg; Folic Acid, 0.45 mg; Biotin, 0.09 mg; Iron, 60 mg; Copper, 8.3 mg; Manganese, 6.7 mg; Zinc, 110 mg; Iodine, 1.3 mg; Selenium, 0.35 mg. ^*d*^Mold-Zap Citrus, Alltech do Brasil Agroindustrial Ltda., Araucária, Brazil. ^*e*^Banox, Alltech do Brasil Agroindustrial Ltda., Araucária, Brazil. ^*f*^ Determined by the sieve method (37).

The extrusion study assessed the effects of LP inclusion on extrusion processing and kibble formation (the diets were extruded on the same day according to a completely randomized design, CRD). For statistical comparisons, the experimental unit (treatment repetition) was used to establish the processing information and samples that were taken every 15 min during a stable extrusion processing. At least four samplings per treatment were obtained to evaluate the processing parameters. Kibble characteristic evaluation was analyzed according to a CRD, and each kibble was considered an experimental unit with 20 repetitions (kibbles) analyzed per treatment.

The ingredients of each diet were weighed individually for each batch and mixed in a horizontal single shaft paddle mixer (450 kg/batch capacity) directly coupled to the high-speed hammer mill fitted with a 0.8 mm size screen sieve where the ingredients were then ground (1000 kg/h capacity, Sistema Tigre de Mistura e Moagem, Tigre, São Paulo, Brazil). Extrusion processing was carried out on a single-screw extruder (MEX- 250, Manzoni Industrial Ltda., Campinas, Brazil) with a production capacity of 250 kg/h, equipped with a differential diameter cylinder preconditioner. The preconditioner shaft speed in the small and large cylinders was set to 60 and 30 rpm, respectively, resulting in an average dough retention time of 180 s in the preconditioner. An extruder screw profile typical for pet foods was used: first section – single flight screw and no steam lock; second section – single flight screw and small steam lock; third section – double flight uncut screw and small steam lock; fourth section – double flight uncut screw and medium steam lock; fifth section – double flight cut cone screw. For operation conditions, steam and water application was only implemented in the preconditioner. The extruder screw was set to 607 rpm for all treatments. The circular extruder die had a single opening (diameter, 8.0 mm; open area, 50.2 mm^2^) and was equipped with a probe to measure the mass temperature at the center of the product flow. The extruder knife speed was set at 940 rpm for all treatments. After extrusion, the extrudates were dried in a dual pass dryer at 110°C for 24 min and coated with poultry fat and liquid palatability enhancer.

The processing parameters were stabilized for the CO diet and kept unchanged for all other treatments to assess the effect of LP inclusion on extrusion traits and kibble macrostructure. Once stable processing conditions were obtained (after ~30 min), the extrusion parameters were recorded and samples from the preconditioner, extruder and dryer collected four times every 15 min per treatment, then stored for later analysis (experimental unit). The specific mechanical energy (SME, kW-h/ton), specific thermal energy (STE, kW-h/ton) and total specific energy (TSE, kW-h/ton) implementations were calculated using the recorded parameters for each treatment repetition and the equation proposed by Riaz (38) and Pacheco et al. (21). Starch gelatinization (39) was also determined for each treatment repetition using enzymatic methods. The mean geometric diameter (MGD) and geometric standard deviation (GSD) of the raw material mixture after grinding were calculated using 100 g of sample in a sieve agitator with a coupled hammer (ABRT 820, Bronzinox, São Paulo, Brazil). An agitation time of 10 min and 12 screen sieve sizes were used: 1.000 mm, 0.841 mm, 0.710 mm, 0.595 mm, 0.500 mm, 0.354 mm, 0.297 mm, 0.250 mm, 0.210 mm, 0.149 mm, 0.125 mm and 0.062 mm plus plates (37). The results were integrated using Granucalc software (Granucalc, Embrapa Suínos e Aves, Concordia, Brazil).

Kibble characteristics and macrostructure were analyzed using kibble samples collected from the dryer. The radial expansion ratio, specific length and piece density were determined by measuring the length, diameter and mass of 20 representative extrudates, as described by Karkle et al. (40). Hardness was analyzed for 20 kibble pieces, stabilized to the same moisture level in an oven at 35°C for 24 h, using a texture analyser (TAX/T2I, Stable Micro Systems, Godalming, UK) equipped with a load cell of 50 kgf (kilogram force) and a cone probe.

#### 2.1.2 Nutrient digestibility of the complete diet and by-product

The *in vivo* study included the analysis of nutrient digestibility, fecal fermentation end-products, glycaemic and insulinemic curves, biogenic amines and nitrogen balance evaluation. These assessments were organized according to a completely randomized block design, containing five diets, two blocks of 20 dogs each, and 4 dogs per diet in each block, totalling eight dogs per diet and 40 dogs in total. The limiting factor for each block was time, due to the impossibility of handling all 40 dogs at the same time in the laboratory. The experimental unit was an individual dog. For the digestibility experiment, dogs were individually housed in 1 × 1 × 1 m stainless steel metabolic cages, purposely made to separate and collect feces and urine.

Nutrients apparent total tract digestibility coefficient (ATTDC) of each dietary treatment was carried out through the quantitative collection of feces, according to FEDIAF recommendations (36), from 40 clinically healthy adult beagle dogs. The health of the animals was previously evaluated by physical examination. Each 17-d digestibility trial included a 12-d food adaptation phase and a 5-d period for the collection of all feces. During the adaptation period, dogs were housed in 1.5 × 4.0 m kennels with a solarium and released daily for 6 hours into a collective playground for exercise and socialization. During the collection period, the dogs were individually housed in 1 × 1 × 1 m stainless steel metabolic cages. The amount of food per dog was calculated to satisfy the metabolizable energy (ME) requirement for adult dogs in maintenance (kcal/d = 110 x BW^0.75^), according to FEDIAF (36), and was offered twice daily (10:00 and 16:00). Offered and refused amounts were weighed and the total intake recorded. Feces were quantitatively collected and weighed at each feeding time and immediately frozen at−20°C. At the end of the digestibility period, feces were thawed, homogenized, pooled per dog, pre-dried in a forced-air oven (MA035, Marconi, Piracicaba, Brazil) at 55°C for 72 h and stored for chemical analysis. The fecal quality was scored on a 0–5 scale (24): 0 = watery liquid feces that can be poured; 1 = soft, formless feces; 2 = soft, unformed stool which assumes the shape of the container; 3 = soft, formed and moist stool that retains shape but leave traces on the floor when picked up; 4 = well-formed and consistent stool; the feces do not leave traces on the floor when picked up; 5 = hard, dry pellets in the form of small and hard mass. During the fecal collection phase, samples of fresh feces were collected within 15 min after excretion (cages were continuously observed) on three consecutive days to measure fecal pH, biogenic amines and the fermentative end-products, short-chain fatty acids (SCFA), lactate and ammonia.

#### 2.1.3 Glucose and insulin postprandial responses

The glucose and insulin postprandial responses of dogs were only determined for the control diet (CO) and the formulation with 100% rice replacement with LP (LP100). A total of 16 healthy adult beagle dogs [9 females and 7 males, all neutered, median age 3 years (IQR 2–5.5), 11.9 ± 1.9 kg BW, with a median BCS of 5 (on a 9-point scale, IQR 5–6) (35)] were enrolled. Postprandial response profiles were evaluated after the digestibility trials following the procedure described by Carciofi et al. (24), with modified blood collection times: 0 (before the meal), 15, 30, 60, 120, 180, 240, 300, 420, 540 and 720 min after the end of the meal. Dogs were conditioned to ingest the entire daily food ration within 10 min in a single meal, meaning that the animals had not eaten anything in the previous 24 h. The time for blood sample collection was started immediately after the end of the meal. Dogs that did not eat all food within 10 min were not tested on that day, but re-tested on the following day. On the day of blood collection, each dog was aseptically catheterized using a peripheral intravenous catheter inserted into the cephalic vein (Angiocath 20 GA x 1.16 in., Becton-Dickinson, Franklin Lakes, NJ, USA). At each timepoint, 3.0 ml blood were collected and divided in two containers. After each blood collection, the catheter was flushed with saline solution to maintain patency. For glucose analysis, 1.0 ml blood was deposited into a tube containing sodium fluoride and EDTA (NaF/Na_2_EDTA, BD Vacutainer), centrifuged at 2000 g for 10 min at 4°C, and the plasma separated into a polypropylene tube. For the insulin test, 2.0 ml blood were deposited in a tube containing anticoagulant (EDTA, BD Vacutainer), centrifuged at 2000 g for 10 min at 4°C and the plasma separated into a polypropylene tube. Glucose and insulin samples were stored at −80°C until analysis. Before collecting the samples, 0.3 ml blood was drawn and discarded to avoid any dilution of the analytes of interest with the saline in the catheter.

#### 2.1.4 Palatability assessment

For the palatability study we used a separate panel of 38 dogs (19 males and 19 females, all neutered) of different breeds and body weight for the palatability assessment of the CO and LP100 diets, conducted according to a CRD. The trial was conducted at Panelis Latin America (Descalvado, São Paulo, Brazil). The “first choice” (first product consumed) and “preferred” product (most consumed) were determined using the two-bowl method (41). Dogs were housed individually. Due to the differences in body weight, the results were calculated as the relative consumption of each diet, and the mean intake of two tested meals was compared.

### 2.2 Chemical analyses

For the chemical composition analysis of ingredients, diets and pre-dried feces, samples were ground in a cutting mill using a 1 mm screen sieve (MA680, Marconi, Piracicaba, Brazil). The chemical composition was determined according to official methods of the AOAC (42) for dry matter (method 934.01), crude fat was determined by acid hydrolysis (method 954.02), and ash content by muffle furnace incineration (method 942.05), crude fiber (method 962.09) and crude protein (method 990.03) were determined using a LECO nitrogen/protein analyser (FP-528, LECO Corporation, Saint Joseph, USA), and total and insoluble dietary fiber by the enzymatic-gravimetric method (method 991.43). Soluble dietary fiber was calculated as total fiber minus insoluble fiber. Organic matter (OM) was calculated as DM minus ash. The gross energy (GE) content of the diets and fecal matter were determined using a bomb calorimeter (model 1261, Parr Instrument Company, Moline, IL, USA). The total starch content was determined using an enzymatic method (43). Amylose and amylopectin were analyzed according to the method by Knutson (44) and expressed as percentage of total starch. To analyse starch cooking upon extrusion, the degree of starch gelatinization was determined using the amyloglucosidase method (39). All analyses were conducted in duplicate and repeated when the variation between duplicates exceeded 5%.

Fecal pH was measured using a digital pH meter (DM20, Digimed Analítica Ltda., São Paulo, Brazil) immediately after collection by mixing 2 g fresh feces with 6 ml ultrapure water. The concentrations of fecal SCFA were analyzed by gas chromatography (GC-2014, Shimadzu Corporation, Kyoto, Japan) according to Erwin et al. (45). Briefly, 10 g feces were mixed in 30 ml 4.2 N formic acid solution (1:3 w/v), precipitated at 4°C for 72 h and centrifuged (5000 g at 15°C for 15 min). Lactic acid was measured according to (46) by mixing 3 g feces with 9 ml milli-Q water and evaluated by colorimetry (Spectrophotometer Quick-Lab, Drake, Sao José do Rio Preto, Brazil). Ammonia was assessed in the extracts prepared for SCFA according to Vieira (47) in a nitrogen distillation system (Tecnal TE-036/1, Tecnal, Piracicaba, Brazil).

The fecal concentrations of biogenic amines were evaluated using 5 g fresh feces, homogenized and added to 7 mL 5% trichloroacetic acid solution, then mixed for 3 min by vortex and centrifuged at 10000 g for 20 min at 4°C (5810R; Eppendorf, Hamburg, Germany), according to Vale (48). The supernatant was filtered through qualitative filter paper, and the residue was extracted twice using 7 then 6 mL 5% trichloroacetic acid solution. Supernatants were then filtered and pooled. The final volume obtained was recorded and frozen. Biogenic amine concentrations in the supernatant were determined by HPLC (HPLC model LC-10AD; Shimadzu Corporation, Kyoto, Japan).

Plasma glucose concentrations were determined using the glucose oxidase test (GOD-ANA, Labtest Diagnóstica S.A., Lagoa Santa, Brazil) in a semiautomated glucose analyzer (Labquest model BIO-2000, Labtest Diagnóstica S.A., Lagoa Santa, Brazil). All analyses were conducted in duplicate and repeated when the variation exceeded 5%. Plasma insulin concentration was measured using a Quantikine ELISA kit (human/canine/porcine insulin immunoassay, R&D Systems, Minneapolis, USA), following the manufacturer's recommendations, in a microplate reader (Biochrom Asys Expert Plus, Biochrom, Cambourne, Cambridge, UK) using a 450 nm filter. The insulin analysis inter- and intra-assay coefficients of variation were 5.7% and 3.6%, respectively. Response profiles were compared by computing the basal, minimum, mean and maximum concentrations, and the time to absolute and incremental peak values (absolute minus basal metabolite value of the animal). The integrated areas under the postprandial glucose and insulin response curves were calculated by numerical integration using the trapezoidal method in R software (49).

### 2.3 Calculations

#### 2.3.1 *In vivo* digestibility of the experimental diets

Apparent total tract digestibility coefficients (ATTDC) of the individual dietary elements of all diets were calculated as follows:

Total fecal collection method (TFC):


$$\begin{array}{c} \text{ATTDC}{\text{X}}_{\text{diet}}\left(\%\right)=\left[\left(\text{total}{\text{X}}_{\text{diet}}–\text{total}{\text{X}}_{\text{faeces}}\right)/\text{total}{\text{X}}_{\text{diet}}\right]\text{x }100 \end{array}$$

where X is the total content of: DM, organic matter (OM), crude protein (CP), acid-hydrolysed fat, total dietary fiber (TDF), or gross energy in the consumed food or feces produced (X_diet_ and X_faeces_, respectively).

#### 2.3.2 *In vivo* digestibility of the lentil pasta by-product

The calculation of the apparent digestibility and metabolizable energy of the LP ingredient was performed using the substitution method as proposed by Matterson et al. (50) and Fortes et al. (51). For the calculation, the inclusion percentage of the ingredient was corrected according to the dry matter content.


$$\begin{array}{c} \text{ATTDCing}=\text{ ATTDCrd}+\;\frac{\text{ATTDCtd}-\text{ATTDCrd}}{\text{Incl}\left(\frac{\text{g}}{\text{kg}}\right)/1000} \end{array}$$

Where:

ATTDCing = Apparent Total Tract Digestibility Coefficient of the ingredient;

ATTDCrd = Apparent Total Tract Digestibility Coefficient of the reference diet (CTRL);

ATTDCtd = Apparent Total Tract Digestibility Coefficient of the test diet (LPS);

Incl(g/kg) = Inclusion level of the ingredient (LP) in the reference diet (CTRL).

#### 2.3.3 Extrusion processing

The SME (kW-h/ton) was calculated for each treatment repetition (experimental unit) using the following equation (38):


$$\begin{array}{c} \text{SME }\left(\frac{\text{kwh}}{\text{t}}\right)=\;\frac{\left(\left(\sqrt{3}\;\times \text{ Voltage x }\left(\text{At}-\text{Av}\right)\;\times \;(cos\text{Fi }\right)\right)}{\text{M}}\; \end{array}$$

Where: Voltage = 220 V; At = torque load working amperage (A); Av = no torque load working amperage (A); cosFi = power factor (0.80); M = mass flow rate from extruder (kg/h).

The STE (kW-h/ton) in the preconditioner and extruder was calculated by mass and energy balance equations according to Riaz (38). The feed, water and steam total input and output mass amounts were determined and used together with the corresponding specific heat values from each component of the system to calculate the amount of heat produced, as described below.

A. Mass Balance:

(1) For Preconditioner


$$\begin{array}{c} {\text{M}}_{\text{r}}+{\text{M}}_{\text{sp}}+{\text{M}}_{\text{wp}}={\text{M}}_{\text{p}}+{\text{M}}_{\text{slp }} \end{array}$$

Where: M_r_ = raw material feed rate (kg/hr); M_sp_ = steam injection into preconditioner (kg/hr); M_wp_ = water injection into preconditioner (kg/hr); M_p_ = preconditioner product flow rate (kg/hr); M_slp_ = steam loss from preconditioner (kg/hr).

(2) For Extruder


$$\begin{array}{c} {\text{M}}_{\text{p}}+{\text{M}}_{\text{we}}={\text{M}}_{\text{sle}}+{\text{M}}_{\text{e}} \end{array}$$

Where: M_p_ = preconditioner product flow rate (kg/hr); M_we_ = water injection into extruder (kg/hr); M_sle_ = steam loss from extruder (kg/hr); M_e_ = product flow rate (kg/hr).

B. Energy Balance:

(1) For Preconditioner


$$\begin{array}{c} {\text{Q}}_{\text{r}}+{\text{Q}}_{\text{sp}}+{\text{Q}}_{\text{wp}}={\text{Q}}_{\text{p}}+{\text{Q}}_{\text{slp}}+{\text{Q}}_{\text{ΣΔhP}}+{\text{Q}}_{\text{LP}} \end{array}$$

Where: Q_r_ = energy flow with raw material (kJ/hr); Q_sp_ = energy flow with steam injection (kJ/hr); Q_wp_ = energy flow with water injection (kJ/hr); Q_p_ = energy flow with flow rate (kJ/hr); Q_slp_ = energy flow with steam loss (kJ/hr); Q_ΣΔ*hP*_ = energy flow to cook starch and protein (kJ/hr) in the preconditioner; Q_LP_ = preconditioner energy loss (kJ/hr).

(2) For Extruder


$$\begin{array}{c} {\text{Q}}_{\text{p}}+{\text{Q}}_{\text{we}}+{\text{Q}}_{\text{SME}}={\text{Q}}_{\text{sle}}+{\text{Q}}_{\text{ΣΔhE}}+{\text{Q}}_{\text{LE}}+{\text{Q}}_{\text{e}} \end{array}$$

Where:

Q_p_ = energy flow with raw flow rate (kJ/hr); Q_we_ = energy flow with water injection (kJ/hr); Q_SME_ = energy flow with specific mechanical energy (kJ/hr); Q_sle_ = energy flow with product flow rate (kJ/hr); Q_ΣΔ*hE*_ = energy flow to cook starch and protein (kJ/hr) in the extruder; Q_LE_ = extruder energy loss (kJ/hr); Q_e_ = energy flow with flow rate (kJ/hr).

The heat (Q) obtained was calculated according to the formula:


$$\begin{array}{c} \text{Q}=\text{m }\times \text{ c }\times \text{ T} \end{array}$$

Where: m = mass; c = specific heat capacity; T = temperature.

The STE was calculated as follows:


$$\begin{array}{c} \text{STE }\left(\frac{\text{kWh}}{\text{t}}\right)=\left(\frac{\text{QΣΔhE}+\text{Qwe}+\text{Qr}+\text{Qwp}+\text{Qsp}+\text{ QΣΔhP}}{\text{Me}}\right)÷3.6 \end{array}$$

The TSE (kW-h/ton) was obtained as the sum of SME and STE.

#### 2.3.4 Kibble macrostructure

For each treatment, the length (l_e_), diameter (d_e_) and mass (m_e_) of 20 extrudates were measured using a Vernier Caliper and a precision scale. The data were then used to calculate the radial expansion ratio (ER), specific length (l_sp_) and piece density (ρ), as described below (40). A die diameter (dd) of 8 mm was used.


$$\begin{array}{c} \text{ER}=\frac{d_{e}^{2}}{d_{d}^{2}}\\ l_{sp}\left(\frac{m}{kg}\right)=\;\frac{l_{e}}{m_{e}}\\ \rho \left(\frac{kg}{m^{3}}\right)=\;\frac{m_{e}}{\pi \;\times \;{\left(\frac{d_{e}}{2}\right)}^{2}\times \;l_{e}} \end{array}$$

#### 2.3.5 Nitrogen balance

Nitrogen levels in the urine samples were determined using a LECO nitrogen/protein analyser (FP-528, LECO Corporation, Saint Joseph, USA). The nitrogen (N) balance was calculated as the difference between the ingested N (Ni) and the N excreted in the feces (Nf) and urine (Nu):


$$\begin{array}{c} \text{Nitrogen balance }\left(\text{mg}/\text{k}{\text{g}}^{0.75}/\text{day}\right)\;=\text{ Ni }\left(\text{mg}/\text{k}{\text{g}}^{0.75}/\text{day}\right)\\ –\left[\text{Nf}\left(\text{mg}/\text{k}{\text{g}}^{0.75}/\text{day}\right)\;+\text{ Nu }\left(\text{mg}/\text{k}{\text{g}}^{0.75}/\text{day}\right)\right] \end{array}$$

### 2.4 Statistical analyses

The results of the extrusion parameters were analyzed according to a CRD with four replications (sampling time) per treatment. The kibble characteristics were evaluated according to a CRD, with 20 experimental units (kibbles) per treatment. Data on apparent digestibility and fecal parameters were analyzed according to a completely randomized block design, with two blocks of 20 dogs and 8 experimental units (dogs) per treatment. All data were tested for normality (Lilliefors test) and homogeneity of variances (using Levene's test), then analyzed by one-way ANOVA. When ANOVA revealed significant differences, we used polynomial contrasts to compare means according to the LP inclusion rate [0% (CO), 33%, 66% and 100%], and orthogonal contrasts (based on Tukey's HSD test) to compare the diets. The fecal score was analyzed by the Kruskal-Wallis test. For analysis of the glycaemic and insulinemic response curves, we calculated the area under the curve (AUC) by numerical integration using the trapezoidal method. Basal, minimum, mean, maximum and AUC data were submitted to Student's *t*-tests. The time to peak in the glycaemic and insulinemic curve analyses were submitted to Mann–Whitney U tests. Data on glycaemia and insulinemia at different time points were submitted to repeated measures ANOVA. Data were evaluated using R Software (version 3.6.1) (49). In the palatability study, the first preference was evaluated using the χ^2^ test, and the food intake ratio by a Student's t-test after checking for normality (using Lilliefors test) and homoscedasticity (*F*-test). Statistical significance was set at *P* < 0.05, while a statistical trend was considered for 0.05 < *P* < 0.1.

## 3 Results

The only difference between diet formulations was the level of rice substitution with LP. Thus, as expected, protein, starch and dietary fiber differed between diets (Table 2), since the protein and dietary fiber content are both lower in rice with respect to LP and the starch content is higher. Ash, acid-hydrolysed fat, crude fiber, gross energy, Ca, P and moisture content were all similar in the CO, LP33, LP66 and LP100 diets.

**Table 2:** Analyzed chemical composition (g/kg, DM basis) of the dog foods and main carbohydrate sources used in the experimental trial.

**Item**	**Experimental diets and ingredients** ^*****a*****^						
	***CO***	***LP33***	***LP66***	***LP100***	***LPS***	***BR***	***LP***
Moisture	88.7	106.8	101.5	101.9	96.9	104.3	89.4
Ash	51.4	53.7	57.2	58.4	44.7	8.4	23.3
Acid-hydrolyzed fat	133.3	131.7	135.5	138.3	120.6	18.8	19.6
Crude protein	296.0	318.7	355.9	388.7	295.7	113.3	299.0
Starch	411.6	376.6	315.1	269.0	418.9	744.1	557.9
Resistant (%)	0.17	0.14	0.20	0.55	0.30	14.07	18.74
Amylose (%)	9.31	9.20	8.50	8.55	10.92	14.89	12.29
Amylopectin (%)	90.69	90.80	91.50	91.45	89.08	85.11	87.71
Amylose: Amylopectin	0.10	0.10	0.09	0.09	0.12	0.17	0.14
Crude fiber	22.8	24.4	25.2	28.1	24.9	12.3	15.6
Total dietary fiber	105.9	110.6	122.4	127.9	102.0	51.1	95.1
Insoluble dietary fiber	98.4	102.4	113.5	117.7	94.7	44.8	87.9
Soluble dietary fiber	7.5	8.1	8.8	10.2	7.3	6.3	7.2
Ca	7.34	7.60	7.58	7.59	6.23	0.06	0.33
P	6.31	6.83	7.16	7.52	5.87	2.00	4.43
Gross energy (MJ/kg)	20.4	20.9	21.1	21.1	20.4	17.7	18.5

^*a*^CO, control diet; LP33, substitution 33% rice with red lentil pasta by-product; LP66, substitution 66% rice with red lentil pasta by-product; LP100, substitution 100% rice with red lentil pasta by-product; LPS, with 70% of the basal diet (CO) and 30% of the lentil pasta by-product; BR, broken rice; LP, red lentil pasta by-product.

During the extrusion procedures, the preconditioner discharge mass temperature was kept at 88.3 ± 1.5°C for all diets (*P* > 0.05) (Table 3). As planned, the throughput and in-barrel moisture level were both similar for all diets (*P* > 0.05), meaning that these parameters did not contribute to any observed differences between dietary treatments. The extruder motor amperage, pressure and mass temperature showed a quadratic trend with LP66 compared to the other diets (*P* < 0.05), indicating a greater resistance to mass flow. This was also observed as an increase in SME with LP66 (*P* < 0.05). However, STE, TSE and the STE/SME ratio were similar for all formulations considered (*P* > 0.05).

**Table 3:** Processing variables, kibble characteristics and macrostructure of the diets.

**Item**	**Experimental diets** ^**1**^				**SEM^2^**	***p*-value**	**Polynomial contrasts**	
	***CO***	***LP33***	***LP66***	***LP100***			***Linear***	***Quadratic***
***Preconditioner***								
Temperature (°C)	88.0	88.3	88.5	88.5	0.373	0.96	0.64	0.88
***Extruder***								
Motor amperage (A)	38.5^a^	38.8^a^	40.0^b^	38.9^ab^	0.182	< 0.01	< 0.05	< 0.05
Pressure (MPa)	24.8^a^	30.0^b^	30.9^b^	29.4^b^	0.653	< 0.001	< 0.001	< 0.001
Mass temperature (°C)	118.5^a^	122.8^ab^	129.8^c^	126.8^bc^	1.265	< 0.001	< 0.001	< 0.05
Throughput (kg/h)	192.9	193.8	198.9	193.8	1.424	0.47	0.45	0.27
Bulk density (g/L)	383.8^a^	402.5^b^	433.3^c^	451.0^d^	6.842	< 0.001	< 0.001	0.95
In-barrel moisture (%)	26.0	25.3	25.4	24.8	0.283	0.55	0.25	0.97
***Energy balance (kW-h/ton)***								
SME^3^	11.1^a^	11.1^a^	13.0^b^	11.7^ab^	0.252	< 0.01	< 0.05	< 0.05
STE^4^	63.5	65.5	71.6	63.9	2.337	0.64	0.67	0.29
TSE^5^	74.6	76.7	84.6	75.6	2.401	0.48	0.54	0.22
STE/SME ratio	58	5.9	5.5	5.5	0.218	0.90	0.49	0.92
***Kibble macrostructure***								
Hardness (N)	84.7^a^	95.9^a^	96.9^ab^	115.6^b^	2.842	< 0.001	< 0.001	0.34
Expansion rate	2.69^b^	2.68^b^	2.51^a^	2.74^b^	0.020	< 0.001	0.63	< 0.001
Piece density (g/cm^3^)	0.49^a^	0.48^a^	0.54^b^	0.50^a^	0.004	< 0.001	< 0.001	< 0.01
Specific length (cm/g)	15.24^b^	15.48^b^	14.81^a^	14.60^a^	0.063	< 0.001	< 0.001	0.08
Starch gelatinization (%)	89.7^a^	90.7^a^	95.9^b^	97.2^b^	0.871	< 0.001	< 0.001	0.80

^1^CO, control diet; LP33, substitution 33% rice with red lentil pasta by-product; LP66, substitution 66% rice with red lentil pasta by-product; LP100, substitution 100% rice with red lentil pasta by-product; LPS, with 70% of the basal diet (CO) and 30% of the lentil pasta by-product. ^2^SEM, standard error of the mean.^3^SME, specific mechanical energy. ^4^STE, specific thermal energy. ^5^TSE, total specific energy. ^*a, b, c, d*^Mean values in the same row not sharing a common superscript letter differ (P < 0.05).

It should be noted that the bulk density underwent a linear increase (*P* < 0.001) as the LP inclusion level increased, which also promoted a linear increment (*P* < 0.001) in kibble hardness and a linear decrease (*P* < 0.001) in the specific length. The expansion rate and the piece density reduced quadratically (*P* < 0.001) with the increase in LP.

Table 4 reports the nutrient intake and ATTDC for the four dietary treatments. All diets were well accepted by the dogs, with no episodes of refusal, vomiting or diarrhea. The inclusion of LP did not affect the intake levels of dry matter, organic matter or acid-hydrolysed fat. As expected, the intake level of CP and TDF increased linearly (*P* < 0.001) as the LP inclusion level rose, whereas starch intake showed a linear decrease (*P* < 0.001).

**Table 4:** Nutrient intake and apparent total tract digestibility coefficient of the different nutrients according to the diets tested.

**Item**	**Experimental diets** ^**1**^				**SEM^2^**	**p-value**	**Polynomial**	
	***CO***	***LP33***	***LP66***	***LP100***			***Linear***	***Quadratic***
***Nutrient intake (g/kg BW** ^0.75^ **/day)***								
Dry matter	24.46	24.07	24.26	24.07	0.102	0.49	0.37	0.79
Organic matter	23.23	22.78	22.72	22.54	0.107	0.14	0.09	0.68
Acid-hydrolysed fat	3.29	3.21	3.26	3.30	0.016	0.24	0.47	0.14
Crude protein	7.43^a^	7.64^a^	8.58^b^	9.31^c^	0.140	< 0.001	< 0.001	< 0.01
Starch	10.33^d^	9.03^c^	7.60^b^	6.44^a^	0.267	< 0.001	< 0.001	0.85
Total dietary fiber	2.66^a^	2.65^a^	2.95^b^	3.06^c^	0.035	< 0.001	< 0.001	0.09
***ATTDC***								
Dry matter	0.847^ab^	0.858^b^	0.855^b^	0.825^a^	0.004	< 0.01	< 0.05	< 0.01
Organic matter	0.872^ab^	0.884^b^	0.876^b^	0.847^a^	0.004	< 0.01	< 0.01	< 0.01
Acid-hydrolysed fat	0.920	0.920	0.922	0.917	0.002	0.89	0.81	0.53
Crude protein	0.764^a^	0.802^ab^	0.808^b^	0.777^ab^	0.005	< 0.05	0.18	< 0.01
Starch	0.999^b^	0.999^b^	0.998^ab^	0.997^a^	0.0002	< 0.01	< 0.001	0.26
Total dietary fiber	0.512^a^	0.580^ab^	0.604^b^	0.546^ab^	0.011	< 0.05	0.12	< 0.01
Gross energy	0.866^ab^	0.880^b^	0.876^b^	0.846^a^	0.004	< 0.01	< 0.05	< 0.01
***Nitrogen balance***								
mg N/kg BW^0.75^/day	67.0	76.7	164.8	102.2	24.7	0.52	0.34	0.39

^1^CO, control diet; LP33, substitution 33% rice with red lentil pasta by-product; LP66, substitution 66% rice with red lentil pasta by-product; LP100, substitution 100% rice with red lentil pasta by-product. ^2^SEM, standard error of the mean. ^*a, b, c, d*^Mean values in the same row not sharing a common superscript letter differ (P < 0.05).

Dry matter, organic matter and gross energy digestibility were lower (*P* < 0.01) in LP100 compared with LP33 and LP66. CP and TDF digestibility were higher (*P* < 0.05) in LP66 with respect to CO. Starch ATTDC was higher (*P* < 0.01) in CO compared with LP100, although the difference has no relevance from the nutritional standpoint (0.999 vs. 0.997).

There was no difference (*P* > 0.05) between groups in the nitrogen balance, with all diets having a positive balance. Table 5 reports the ATTDC for the ingredient alone (LP), calculated according to the methods reported by Matterson et al. (50). The results indicate the ingredient has high apparent nutrient digestibility by the dogs.

**Table 5:** Apparent total tract digestibility coefficient (average ± SD) of the different nutrients and energy of red lentil pasta by-product calculated according to Matterson et al. (50).

	***ATTDC***						
	**Dry matter**	**Organic matter**	**Acid-hydrolysed fat**	**Crude protein**	**Starch**	**Total dietary fiber**	**Gross energy**
LP^*a*^	0.939 ± 0.027	0.938 ± 0.026	0.811 ± 0.055	0.877 ± 0.053	0.998 ± 0.002	0.765 ± 0.105	0.938 ± 0.036

^*a*^LP, red lentil pasta by-product.

The increases in LP inclusion correlated with a linear increase (*P* < 0.05) in feces production and a linear decrease (*P* < 0.05) in fecal DM and pH (Table 6). However, despite the increase in moisture content, the fecal score remained similar at a median value of 4 (well-formed, consistent stools) for all diets throughout the digestibility trial. During the adaptation period, two dogs in the LP100 group and one dog in the LP66 group had loose feces (fecal score of 2 or 3) for five to seven days. No more signs of diarrheic stools occurred thereafter, suggesting the potential need for an adjustment period at these LP inclusion levels.

**Table 6:** Fecal characteristics and fermentation products of dogs fed diets with different inclusion of red lentil pasta by-product.

**Item**	**Experimental diets** ^**1**^				**SEM^2^**	***p*-value**	**Polynomial contrasts**	
	***CO***	***LP33***	***LP66***	***LP100***			***Linear***	***Quadratic***
***Feces characteristics***								
g/kg BW^0.75^/day (as fresh matter)	10.9^a^	10.2^a^	11.3^a^	14.6^b^	0.43	< 0.001	< 0.05	0.07
Fecal DM (g/kg)	353.6^c^	334.1^bc^	311.3^ab^	288.9^a^	5.20	< 0.001	< 0.05	0.18
Fecal score^3^	4.0	4.0	4.0	4.0	-	-	-	-
pH	6.24^b^	6.30^b^	6.19^ab^	5.96^a^	0.04	< 0.01	< 0.05	0.27
***Fermentation products (mMol/g of DM)***								
Acetic acid	275.2^a^	284.1^a^	307.5^ab^	376.7^b^	12.1	< 0.05	< 0.01	0.13
Propionic acid	159.8^a^	181.2^ab^	214.8^b^	290.8^c^	10.5	< 0.001	< 0.001	< 0.05
Butyric acid	66.9	64.8	57.6	64.4	1.97	0.40	0.33	0.23
Total acetic, propionic, butyric acids	503.3^a^	530.2^a^	578.1^a^	718.4^b^	21.5	< 0.001	< 0.001	0.07
Valeric acid	6.64^ab^	4.99^a^	7.45^ab^	14.5^b^	1.26	< 0.05	< 0.05	0.06
Isobutyric acid	15.4	15.12	12.5	13.1	0.49	0.07	0.17	0.10
Isovaleric acid	27.6^ab^	28.4^b^	22.2^ab^	21.5^a^	0.97	< 0.05	< 0.05	0.26
Total bVFA	43.0^ab^	44.0^b^	34.7^ab^	33.9^a^	1.49	< 0.05	< 0.05	0.25
Total VFA	553.0^a^	579.2^a^	620.3^a^	766.8^b^	21.8	< 0.001	< 0.05	0.35
Ammonia (mMol/kg of DM)	350.6	361.1	316.1	318.2	10.9	0.37	0.20	0.50
Lactate (mMol/kg of DM)	4.98^a^	5.22^ab^	6.34^bc^	7.24^c^	0.22	< 0.001	< 0.01	0.54

^1^CO, control diet; LP33, substitution 33% rice with red lentil pasta by-product; LP66, substitution 66% rice with red lentil pasta by-product; LP100, substitution 100% rice with red lentil pasta by-product. ^2^SEM, standard error of the mean. ^3^Fecal score on a 0–5 scale (24): 0 = watery liquid feces that can be poured; 1 = soft feces, formless; 2 = soft, unformed stool which assumes the shape of the container; 3 = soft, formed and moist stool that retains shape but leave traces on the floor when picked up; 4 = well-formed and consistent stool; the feces do not leave traces on the floor when picked up; 5 = hard, dry pellets in the form of small and hard mass. Calculated as the median of all of the observations. ^*a, b, c*^Mean values in the same row not sharing a common superscript letter differ (P < 0.05).

The increasing levels of LP inclusion caused a linear increase in acetic acid (*P* < 0.01), propionic acid (*P* < 0.001), valeric acid, total VFA (*P* < 0.05) and lactate (*P* < 0.01) in the feces. By contrast, there was a linear decrease in isovaleric acid and total branched volatile fatty acids (bVFA) (*P* < 0.05). Fecal levels of butyric acid, iso-butyric acid and ammonia did not differ between dietary groups (*P* > 0.05)

Serotonin and agmatine biogenic amines were below the detection limit (0.4 mg/kg of DM) in all fecal samples analyzed. Putrescine, phenethylamine and tryptamine did not vary (*P* > 0.05) between dietary groups (Table 7). Increasing LP inclusion led to a linear increase in cadaverine, histamine, spermidine (*P* < 0.001) and tyramine (*P* < 0.01) concentrations. Spermine presented a quadratic increase from CO to LP33, but levels then dropped significantly in the LP100 diet (*P* < 0.01).

**Table 7:** Fecal biogenic amines concentration (mg/kg of dry matter) in dogs fed diets with different inclusion of red lentil pasta by-product.

**Item**	**Experimental diets** ^**1**^				**SEM^2^**	***p*-value**	**Polynomial contrasts**	
	***CO***	***LP33***	***LP66***	***LP100***			***Linear***	***Quadratic***
Putrescine	478.3	757.6	688.5	832.5	61.2	0.20	0.51	0.82
Cadaverine	192.8^a^	512.8^ab^	777.1^ab^	1163.3^b^	103.7	< 0.01	< 0.001	0.74
Tyramine	19.2^a^	37.5^a^	85.6^ab^	188.2^c^	20.1	< 0.05	< 0.01	0.19
Histamine	8.1^a^	9.4^a^	35.0^a^	118.6^b^	11.5	< 0.001	< 0.001	< 0.05
Spermidine	65.7^a^	79.7^ab^	105.6^bc^	117.0^c^	5.7	< 0.01	< 0.001	0.88
Phenethylamine	5.3	7.8	6.6	8.2	1.2	0.86	0.61	0.96
Spermine	14.8^a^	90.9^b^	64.6^ab^	25.4^a^	9.2	< 0.01	0.97	< 0.01
Tryptamine	23.0	28.7	13.1	26.6	3.2	0.33	0.68	0.37

^1^CO, control diet; LP33, substitution 33% rice with red lentil pasta by-product; LP66, substitution 66% rice with red lentil pasta by-product; LP100, substitution 100% rice with red lentil pasta by-product; LP, red lentil pasta by-product [as ingredient calculated using (50) from the results of the LPS diet]. ^2^SEM, standard error of the mean. ^*a, b, c*^Mean values in the same row not sharing a common superscript letter differ (P < 0.05).

**Figure 1:**
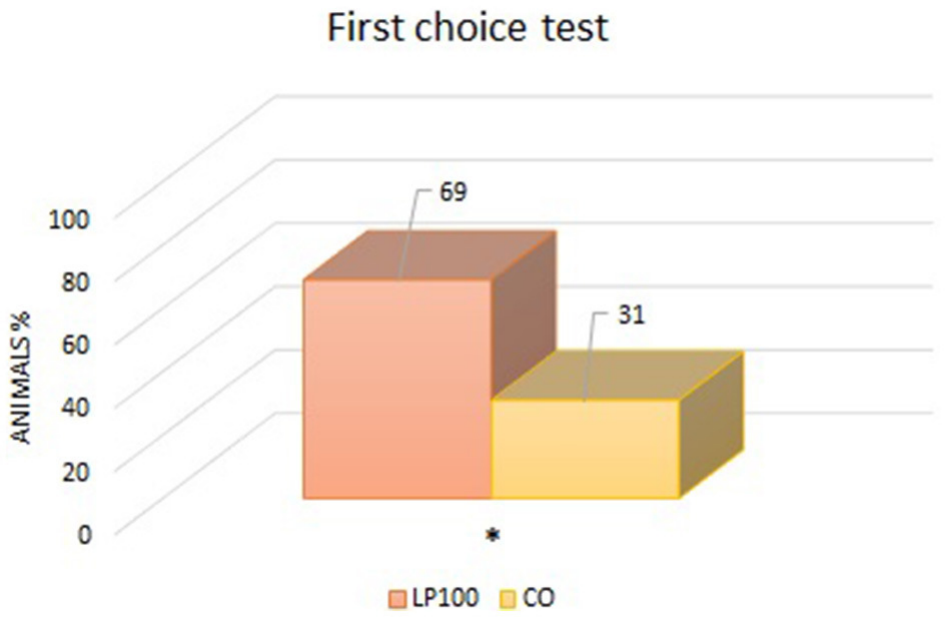
Analysis of the percentage of animals which consumed first one diet proposed compared to another (LP100 vs. CO). *p < 0.05.

**Figure 2:**
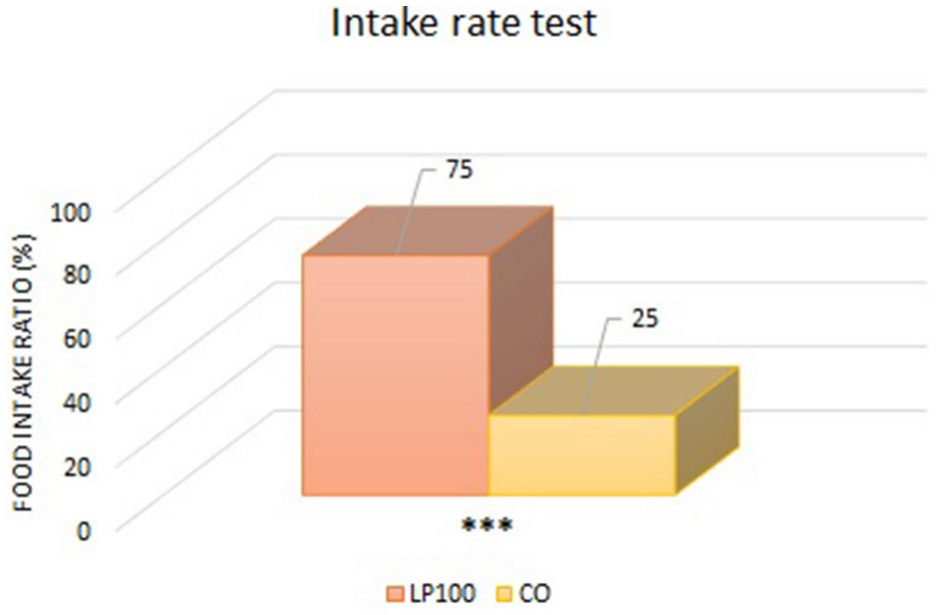
Analysis of the food consumed by the animals as a percentage of the total consumed (LP100 vs. CO). ***p < 0.001.

Two animals were excluded from the palatability study due to underconsumption. All other dogs ate normally without any signs of vomit, with adequate qualities of feces produced. From the “first choice” analysis, we found an overall preference for the LP100 diet (*P* < 0.05) (Figure 1), irrespective of dog breed. The “intake rate” analysis (percentage of the total feed consumed) confirmed the preference for the LP100 diet (*P* < 0.001), once again irrespective of breed (Figure 2).

The glycaemic and insulinemic postprandial response curves showed significantly higher (*P* < 0.05) basal, minimum and mean glucose concentrations following consumption of the CO diet compared with LP100. There was also a significant trend for a higher maximum glucose concentration with the CO diet (*p* = 0.07) (see Table 8).

**Table 8:** Basal, minimum, mean, maximum, time to peak, and area under the curve (AUC) of glucose of dogs fed diets formulated with broken rice (CO) or red lentil pasta by-product (LP100).

**Item**	**Experimental diets**		***p*-value**
	***CO***	***LP100***	
Basal glucose (mg/dL)	83.6 ± 5.3	78.2 ± 4.0	< 0.05
Minimum glucose (mg/dL)	79.8 ± 5.6	73.9 ± 3.9	< 0.05
Average glucose (mg/dL)	89.6 ± 7.7	81.6 ± 2.6	< 0.05
Maximum glucose (mg/dL)	101.4 ± 12.1	91.7 ± 5.5	0.07
Time to peak (min)	150 (60-180)	180 (165-240)	< 0.05
**AUC (mg/dL/h)**			
0-60'	86.6 ± 5.3	79.6 ± 3.8	< 0.05
60-120'	94.4 ± 9.5	83.0 ± 4.7	< 0.05
120-300'	276.8 ± 57.1	256.5 ± 15.8	0.36
300-720'	593.2 ± 92.6	562.3 ± 17.1	0.38
0-720'	1080.9 ± 104.0	981.5 ± 30.9	< 0.05

Values expressed as average ± standard deviation or median (interquartile range).

**Figure 3:**
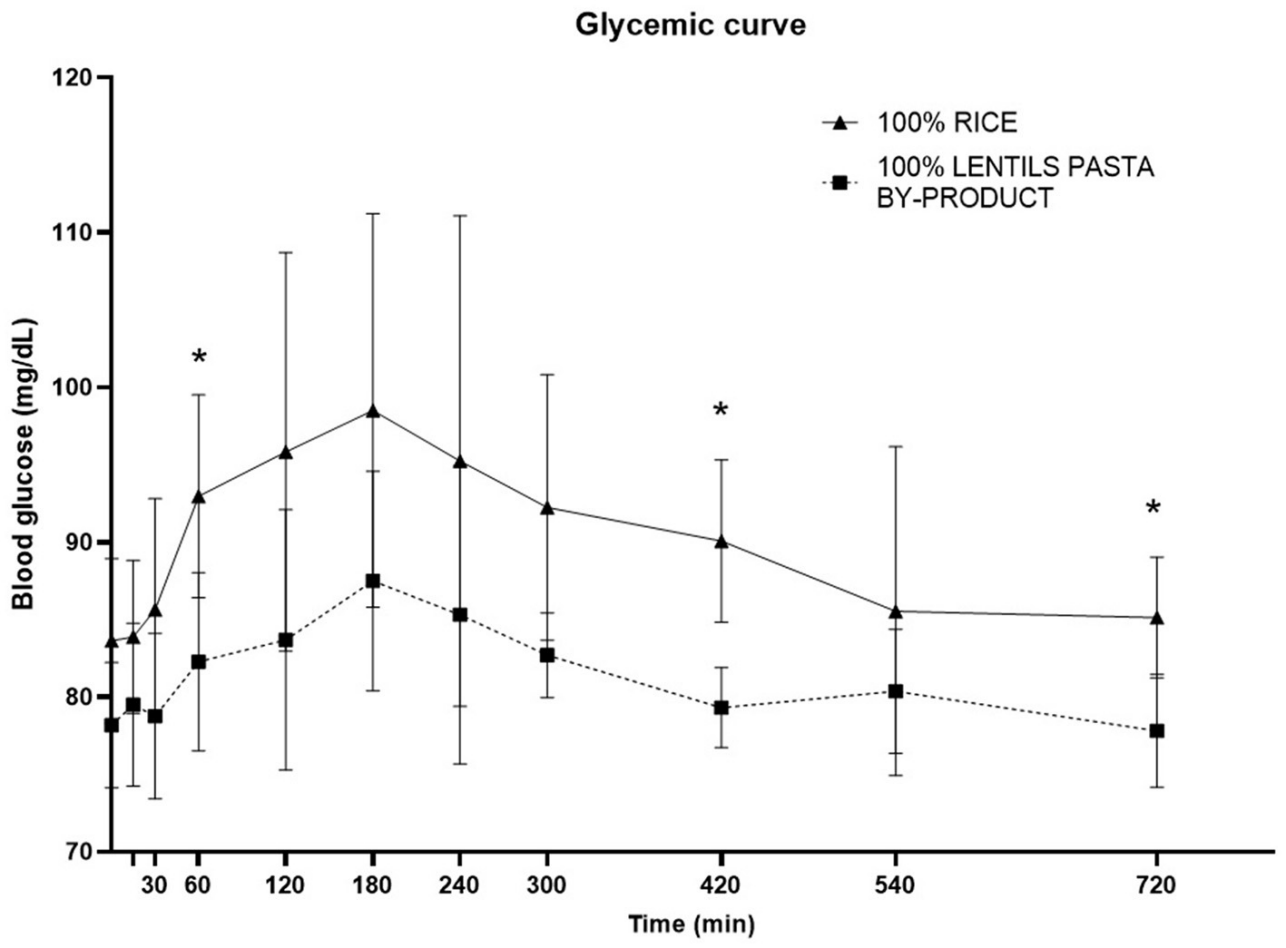
Postprandial responses of plasma glucose (mg/dL) according to the time of adult dogs fed CO vs. LP100 diets. *p < 0.05.

The time to glucose peak was slower following consumption of the LP100 diet (median, 180 min) compared with CO (median, 150 min, *P* < 0.05). The AUC 0-60', AUC 60-120' and total AUC of the glucose response were all smaller (*P* < 0.05) in dogs fed LP100. Glucose concentrations were significantly lower (*P* < 0.05) at 60', 420' and 720' in dogs fed LP100 (Figure 3). Similarly, the incremental glucose curve showed a delayed glucose peak as well as a lower total AUC (*P* < 0.05) in dogs receiving LP100, while the maximum incremental glucose concentration was greater (*P* < 0.05) in the CO diet (18.4 mg/dL) compared with LP100 (9.3 mg/dL; data not shown).

**Figure 4:**
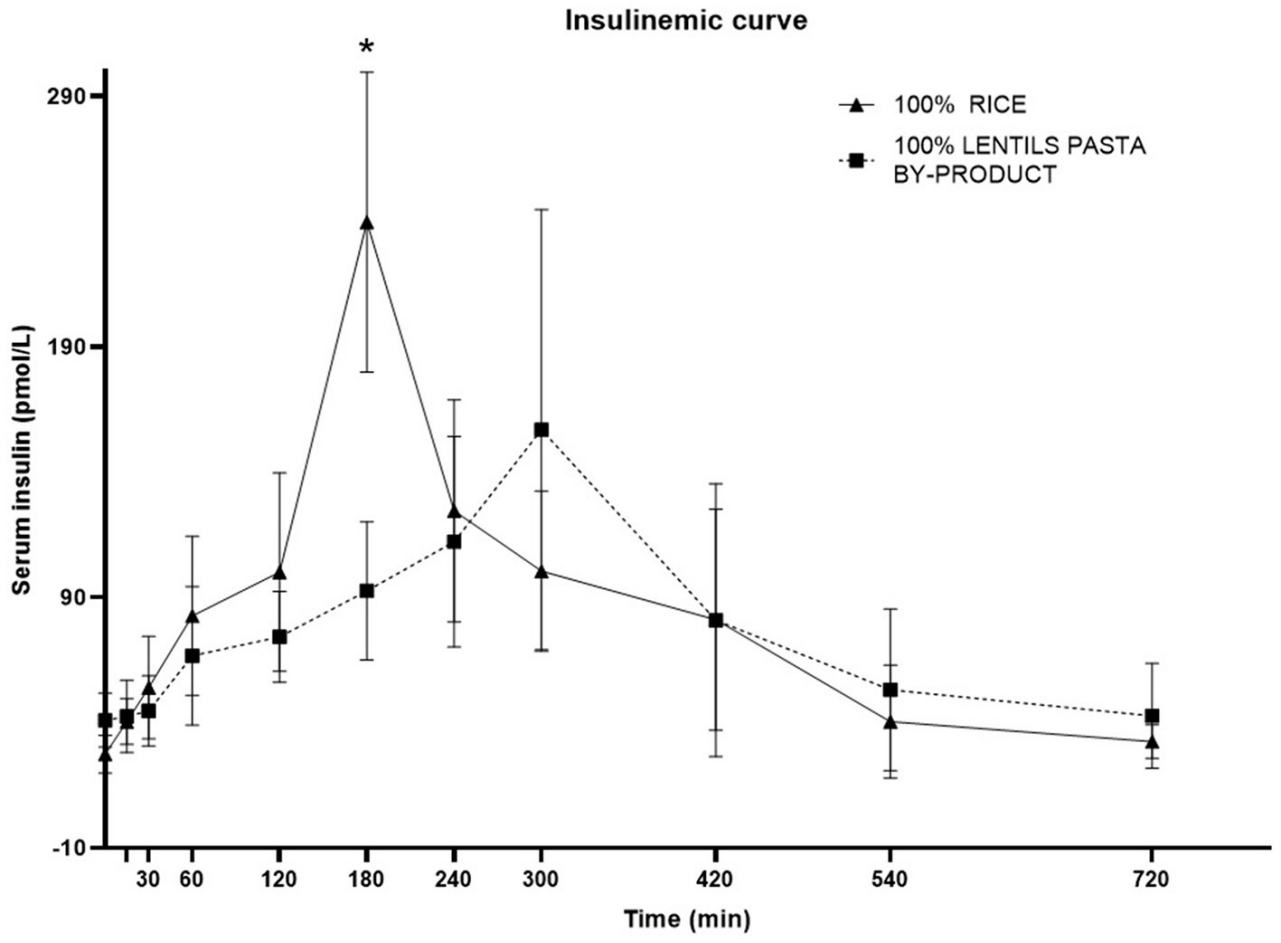
Postprandial responses of plasma insulin (mg/dL) according to the time of adult dogs fed CO vs. LP100 diets. *p < 0.05.

The plasma insulin response curve for dogs fed LP100 showed a lower and delayed peak (300 min, IQR 240–300) compared with those fed CO (180 min; IQR 180–180; *P* < 0.05). The 60-240' AUC was also lower (*P* < 0.05) in LP100, showing a constant increase during the first 4 h after the meal (Figure 4). The basal insulin concentration differed between treatments (*P* < 0.05), being higher in dogs fed the LP100 diet (40.9 ± 10.7 mg/dL) compared with CO (27.3 ± 7.6 mg/dL) (see Table 9). Both groups returned to their respective basal insulin levels (LP100: 42.7 ± 20.9 mg/dL; CO: 32.4 ± 6.7 mg/dL) at time 720', possibly the result of a slower decrease in the curve for the LP100 diet. The incremental plasma insulin response curve showed even more conspicuous results between the two groups. Specifically, the LP100 group had lower incremental insulin levels at 30', 60', 120' (*P* < 0.05) and 180' (*P* < 0.01) compared with CO. Likewise, the incremental plasma insulin curve for dogs fed the LP100 diet showed a lower and delayed peak (median, 300 min) compared with CO (median, 180 min; *P* < 0.05). In addition, the incremental insulin AUC 0-60' and AUC 60-240' were smaller (*P* < 0.01) in the LP100 group in contrast with CO, for which a sharp increase was observed (data not shown).

**Table 9:** Basal, minimum, mean, maximum, time to peak, and area under the curve (AUC) of insulin of dogs fed diets formulated with broken rice (CO) or red lentil pasta by-product (LP100).

**Item**	**Experimental diets**		***p*-value**
	***CO***	***LP100***	
Basal insulin (mg/dL)	27.3 ± 7.6	40.9 ± 10.7	< 0.05
Minimum insulin (mg/dL)	23.6 ± 2.9	28.7 ± 8.9	0.15
Average insulin (mg/dL)	78.0 ± 24.3	73.7 ± 21.6	0.71
Maximum insulin (mg/dL)	189.5 ± 83.0	166.8 ± 79.5	0.58
Time to peak (min)	180 (180-180)	300 (240-300)	< 0.05
**AUC (mg/dL/h)**			
0-60'	54.4 ± 13.1	45.8 ± 14.0	0.23
60-240'	393.0 ± 13.1	263.6 ± 90.9	< 0.05
240-420'	268.9 ± 110.8	372.2 ± 181.4	0.19
420-720'	217.4 ± 91.4	277.6 ± 147.5	0.35
0-720'	972.2 ± 281.0	969.6 ± 351.7	0.99

Values expressed as average ± standard deviation or median (interquartile range).

## 4 Discussion

Research interest in the use of pulses in the pet food industry is growing fast, but data on the extrusion processing and kibble formation of dog food containing this ingredient is scarce (52), and no studies have been performed to date with respect to lentils. Despite this lack of scientific knowledge, commercial dry pet food formulations containing legumes or tubers instead of cereals as the main carbohydrate source in food (i.e., the grain-free diets) are becoming increasingly popular, with the pet food industry claiming superior health and functional properties of these formulations. Thus, studies are required to understand the effect of their inclusion on the extrusion parameters and variables involved in kibble formation and their characteristics, as very few studies have evaluated this aspect (15, 24–26).

Since even small variations in processing conditions may influence extrusion variables and product quality (53), all operational variables (feed rate, preconditioner temperature, screw configuration and speed, die design, in-barrel moisture and knife speed) were kept constant in this study in order to evaluate the effects of the gradual inclusion of LP on the output parameters studied. Therefore, any measured variation was related to the food formulation. We observed an increase in the extruder motor amperage and SME application when producing the LP66 diet compared with the other diets. The inclusion of LP in the formulation (LP33, LP66 and LP100) also resulted in an increase in extruder pressure compared with the CO diet, and the die temperature increased linearly as the LP inclusion level increased. These factors need to be considered when producing kibble using pulses as the higher fiber and protein content as well as the lower concentration of starch may increase the resistance to mass flow.

The linear increase in kibble bulk density with increasing LP inclusion level may also be explained by the differences in protein, starch and dietary fiber content, in particular the latter (19). Although the expansion rate was similar for all diets (with the exception of LP66), the hardness increased linearly with increasing LP level, probably due, once again, to the reduction in starch and rise in fiber with respect to CO (54, 55). In a previous study, the inclusion of another legume (pea) into rice-based products led to a reduction in the expansion ratio (56). Similarly, increasing fava bean inclusion (0, 10, 20 and 30%) in a dog food formulation led to a linear reduction in expansion ratio and a linear increase in piece density and hardness (52). In addition, extruded chickpeas were reported to have a lower expansion ratio but higher bulk density and hardness compared with cereals (maize and sorghum) when processed using the same extrusion conditions (31, 57).

Although higher fiber inclusion has been related to a reduction in starch gelatinization (19), all the diets in the present study showed a high degree of starch gelatinization (i.e., starch cooking), independent of LP inclusion. Furthermore, the LP inclusion did not appear to affect the amylose:amylopectin ratio.

Despite the differences in nutritional composition between the diets due to the different chemical composition of rice vs. LP, no differences were observed in DM or fat intake. The digestibility of nutrients in the CO diet was similar to that of the diets including LP, with the exception of CP and TDF in LP66. In fact, when the replacement of rice with LP was only partial, the digestibility of these nutrients increased according to a quadratic trend. That said, the digestibility of DM, OM and GE were lower in LP100 with respect to LP66. Similarly, in another trial when legumes (pea, chickpea and fava beans) completely substituted rice, the nutrient digestibility was lower compared with a 50% substitution (58). The decrease in nutrient digestibility following the addition of a carbohydrate source rich in fiber has already been reported (24). Nonetheless, the inclusion of LP in the dog food formulation appeared to be adequate in term of both protein utilization and nitrogen balance.

Nutrient digestibility values were lower here compared with other studies on pulses (15, 26, 58, 59). When investigating the possible causes for this discrepancy, the poultry by-product ingredient used in our formulation was identified as the main culprit. Microscopic analysis of the ingredient revealed its contamination with chicken feet and feathers (sources of indigestible collagen and keratin). Further confirmation of this contamination in the poultry by-product was the *in vitro* digestibility determination of the crude protein using the methods by Hervera et al. (60). In fact, the *in vitro* digestibility showed a lower crude protein digestibility of the poultry by-product used in the diets of this study (60.96%) compared with a different batch of the same ingredient (90.56%). Due to this unpredictable occurrence also the level of calcium and phosphorus resulted lower compared with the expected recipe, but still above the minimum requirements for dogs (36). Despite this unfortunate circumstance, the comparison of diets was not affected as the level of inclusion of the poultry by-product was the same in all diets tested.

The level of starch digestibility in this study was high (≥ 99.7% in all diets) compared with other studies using lentils in dog food preparations (24, 61); however, this could be due to the fact that the LP ingredient had previously been extruded in the pasta making process using a low shear processing method, and thus pre-cooked, which would have increased its subsequent starch digestibility in the dog food formulations. Several recent publications have correlated dilated cardiomyopathy in dogs with the consumption of pulses and grain-free diets (62–64). However, many factors need to be studied before conclusions are drawn (65). A recent study (61) explored the levels of blood taurine in dogs fed six diets formulated using different legumes (one of which was red lentils) as a substitution for rice. After seven days, the plasma concentration of taurine remained within normal ranges for all diets considered. Although long-term studies are needed, if the diet supplies sufficient levels of taurine precursors (methionine and cysteine) and the bioavailability of these amino acids is adequate, taking into consideration the lower digestibility of grain-free diets due to their higher fiber content, then the diet should be of minimum concern (66–68). Moreover, dogs may present other factors associated with dilated cardiomyopathy (69), thus caution should be taken before automatically correlating the disease with diet.

Fecal quality is an important parameter to evaluate in the assessment of pet food quality. In this study, increasing LP inclusion levels correlated with a linear increase in fecal bulk and a linear decrease in fecal DM and pH. Indeed, these findings were expected as the TDF intake also increased linearly with LP inclusion level. Comparable results were found in dogs fed other grain-free diets, such as those based on peas and lentils (24), a combination of peas, potatoes and tapioca (26) or chickpeas, fava beans and peas (58). The increase in the TDF fraction, especially insoluble fiber, accounts for the rise in fecal output since it is poorly fermented in the large intestine, and this may lead to increased peristalsis and water retention capacity (70). Despite the increase in fecal bulk and decrease in fecal DM, the median fecal score for all diets remained at 4 (optimal, well-formed feces) throughout the digestibility trial. The decrease in fecal pH is generally associated with the fermentation of soluble fiber, which may possibly inhibit the growth of pathogenic bacteria. Similarly to Carciofi et al. (24), this study found total rice substitution with LP (LP100) to lower the fecal pH with respect to the CO diet. It is important to note that only dehulled lentils are used to make the red lentil pasta; thus the (fiber-related) effects of this by-product may have been milder than if whole lentils had been used in the dog food formulation, as a large proportion of TDF resides in the hull.

The colonic microbiota is responsible for the fermentation of dietary fiber. These fermentation processes performed by these microorganisms produce a series of metabolic products, such as volatile fatty acids (VFA), gases (H_2_, ammonia and methane) and lactate (71, 72). Since the TDF intake rose with increasing LP inclusion, an increment in fecal VFA content was also expected, as confirmed. In fact, the linear increase in lactate and total VFA and the reduction in fecal pH with increasing LP inclusion are all indicators of corresponding changes in colonic bacterial fermentation. Specifically, acetic, propionic and valeric acids increased linearly with the rise in rice substitution with LP. The production of VFA (e.g., acetate, propionate and butyrate) can provide energy for the epithelial cell growth (73). In fact, the production of SCFA through microbial fermentation in the colon not only accounts for up to 7% of metabolic energy provision in dogs but it is also important for normal colonic absorptive processes (74). Moreover, VFA production reduces the pH, encouraging beneficial bacteria growth whilst inhibiting harmful bacteria development (75). Butyrate is involved in numerous physiological functions, including cell differentiation, anti-inflammatory processes and enhanced colonic immune response, and it is the main energy source for enterocytes (76–78). However, not all sources of fermentable fiber result in an increase in butyrate, as previously observed for beet pulp (75, 79). Indeed, despite the rise in total VFA observed in this study, LP inclusion did not affect the fecal concentration of butyric acid. Nonetheless, it is important to remember that propionic and lactic acid can also be used as energy sources for hepatocytes, while acetic acid can be an energy substrate for peripheral tissues (80). In addition, in human physiology, propionate appears to exert a role in improving glucose tolerance as well as insulin sensitivity (81). Moreover, an *in vitro* study showed propionate to exert an anti-inflammatory effect on the gut and antioxidant properties on the blood-brain barrier (82). For these reasons, increased fecal propionate concentrations should be considered potential biomarkers of improved gastrointestinal functionality in dogs (83).

Biogenic amines are formed by commensal microorganisms in the gut, mainly through the decarboxylation of amino acids derived from unabsorbed endogenous or undigested protein (84–87). As a consequence, the presence of undigested amino acids may promote the proliferation of microorganisms which use them as energy sources (88). The functions and benefits of these amines, and concerns related to their production in the gut, are still being debated since they can exert both positive and negative effects depending on their concentration (87). However, the threshold levels at which beneficial or harmful effects occur has yet to be defined in humans, let alone in pets.

Here, we observed no significant differences in putrescine, phenylethylamine or tryptamine between dietary treatments. In other species, putrescine has been suggested to exert a protective effect on the intestinal mucosa (89), and play a role in DNA, RNA and protein synthesis (90) and small intestine development (91); putrescine can also be used as an energy source in the gut (92). However, in humans, excessive putrescine supplementation can be toxic and reduce growth, show a reduced anticarcinogenic activity and induce impaired spatial learning and memory (87).

The increase in LP inclusion in the dog food formulations led to a linear increase in cadaverine, tyramine, histamine and spermidine; while a quadratic increase was observed for spermine at LP33. Cadaverine is involved in several cellular processes, exerting a protective effect over epithelial cells (93–95), while spermine plays an essential role in cell growth, differentiation and modulation of the ion channel receptors, mucosal repair and healing processes (96–100). Spermidine, besides promoting adipogenesis, shows similar effects to spermine (87). Histamine interacts with various cellular targets (e.g., epithelial or smooth muscle cells) and regulates gastrointestinal functions (such as gastric acid production, intestinal motility and mucosal ion secretion) via histamine receptors disseminated throughout the gastrointestinal tract (101, 102). Tyramine stimulates glucose transport in adipocytes through its oxidation by monoamine oxidase (87). However, excessive oral ingestion of spermine and spermidine can have several effects, including the induction of allergic-like reactions due to modulation of the gastrointestinal epithelial barrier, mucosal damage and morphological changes in villous height (87). Similarly, an excess in histamine in the diet has been related to food poisoning with allergic-like reactions (103, 104), which may be enhanced in the presence of other biogenic amines such as cadaverine, putrescine or tyramine (105). In mice, high concentrations of tyramine increased susceptibility to enteric infection by increasing the adherence of *E. coli* O157:H7 to the caecal mucosa in a concentration-dependant manner (87).

Since the biological consequences of biogenic amine production in the gut by the resident microbiota are largely unknown, it is difficult to predict whether their increase could lead to beneficial or adverse effects (87). In another study, an increase in biogenic amine production was associated with the incidence of diarrhea in weaning pigs fed high protein diets (106). Considering this, it is important to note the lack of any change in stool quality or fecal score in this study as fecal amine levels increased in relation to LP inclusion level. Indeed, the gradual increase in CP and reduction in starch as LP inclusion increased were associated with a linear increase in most of the biogenic amines. As such, the commensal bacterial community in the canine gut, responsible for their formation, may also have been changed.

Palatability is an essential aspect of any pet food formulation. If an ingredient is not accepted by the target animal species, it is of little use for the industry, independently of its nutritional properties. In the current study, the total rice substitution with LP increased the food's acceptability with respect to the CO diet. In another dog food trial in which chickpea, pea and fava beans replaced rice as the carbohydrate source, the animals' acceptance of the product was similar or lower compared with the control (rice based) diet (58). Similarly, a study comparing a traditional grain-based diet with a legume-plus-tuber-based diet found no differences in the dogs' preferences (26). In another similar trial, dogs preferred the control diet with respect to that formulated with a 10 or 30% rice replacement with fava bean, while first choice and food intake at the 20% inclusion level were comparable to control diet (15). Thus, our results are in contrast with these previous findings, although the diets tested here (LP100 and CO) differed in kibble hardness and bulk density, since the extrusion parameters were kept constant during the processing. Moisture content can also influence palatability, but this variable was kept constant in our experimental diets. The palatability of a food relates to both ingredient composition and physical characteristics, making its evaluation complex (107). According to Félix et al. (108), the kibble size, shape, density, humidity level and constituent particle size all influence its palatability. Thus, these parameters were evaluated in the present study. The dry matter, radial expansion and specific gravity of the different kibble formulations did not differ (*P* > 0.05). Only the specific length differed, which was shorter in LP100 (*P* < 0.01). Therefore, palatability differences may be attributed to the characteristics of LP, while acknowledging that the previous low shear extrusion of the ingredient and the fact that the lentils were dehulled may have contributed to the organoleptic attributes of the ingredient.

A dog's glycaemic and insulinemic response to a carbohydrate source is of great importance, especially in those requiring a low, prolonged response. Indeed, obesity and insulin resistance are a significant problem in the canine population (109, 110). To date, only a handful of studies have evaluated glucose and insulin responses in dogs fed different kinds of carbohydrate source (24, 58, 111–114). In the present study, dogs in the LP100 group showed reduced glycaemia and a lower and delayed insulinemic peak with respect to CO, in accordance with a similar study (24). Likewise, dogs fed pulses alone (including lentils) or included in a complete diet showed low glycaemic and insulinemic postprandial responses (115). This is also in agreement with the findings of Adolphe et al. (113) and Rankovic et al. (114), who found that a diet containing pulses led to delayed and prolonged postprandial glucose and insulin responses, lower peak concentrations and a longer time to peak.

Starch is generally considered the dog food nutrient influencing postprandial glycaemic and insulinemic the most (116). Moreover, several factors influence the digestion and absorption of starch sources, which may be intrinsic (e.g., amylose to amylopectin ratio) or extrinsic (processing-related) (117). Other dietary nutrients (e.g., protein, fat and dietary fiber) do not seem to affect the glucose profiles in dogs as much as starch content (19, 116). As the amylose to amylopectin ratio varies between different carbohydrate sources, according to the botanical origin of the starch (118), this parameter was also tested in the experimental diets. Indeed, legumes generally present a higher amylose content compared with cereals; they should, therefore, induce a lower glycaemic response as amylose is less susceptible to enzymatic hydrolysation than amylopectin (119, 120). Although a lower glycaemic and insulinemic response was observed here for the LP100 diet with respect to CO, the levels of amylose and amylopectin and the amylose to amylopectin ratio were unexpectedly similar between the diets. The factors which may have affected the results include the different starch, protein and fiber content of the two diets. Differences in the processing technique may also have affected the results since the LP ingredient had previously been subjected to low-shear extrusion during pasta production, possibly leading to a change in its starch properties. Furthermore, we cannot rule out a possible effect of the higher VFA concentrations in LP100 as both propionate and acetate have a positive effect on glucose tolerance and insulin sensitivity (81, 121, 122).

It is also interesting to note that the LP100 diet led to a lower blood glucose response (both basal and mean concentrations throughout the trial period) compared with the CO diet, although no differences were found between the dogs prior to the trial. This was also reflected in the constantly higher blood insulin concentration in the LP100 group, both before and 12 h after the meal, meaning that the dogs in this group had a steadier and smoother insulin curve compared with those in the CO group. These findings have important implications for dogs requiring delayed and prolonged responses in postprandial blood glucose and insulin levels.

## 5 Conclusions

The substitution of rice with of a red lentil pasta by-product influenced extrusion processing, with higher resistance to mass flow and mechanical energy application, resulting in kibble that were less expanded and harder. These effects will need to be considered when establishing the ideal processing conditions for a LP-based dog food. The substitution did not reduce the apparent digestibility or metabolizable energy of nutrients at the 33 or 66% inclusion levels, and it increased the level of fermentation by-products in the feces (VFA and biogenic amines), indicating the potential benefits of LP on intestinal health due to its high content of fermentable fiber. The higher TDF content of LP with respect to rice and its higher protein and relatively lower starch content might explain the lower post-prandial glucose and insulin responses in dogs, features which would make it particular interesting for use in diets designed to produce a low glycaemic response. Finally, palatability was enhanced in the formulation containing the red lentil pasta by-product, further supporting its use as a suitable ingredient in dog food.

## Data availability statement

The raw data supporting the conclusions of this article will be made available by the authors, without undue reservation.

## Ethics statement

The animal study was approved by the Bioethics Committee (CEUA) of the Universidade Estadual Paulista (UNESP) – Jaboticabal Campus (Brazil) (prot. n. 1501/21). The study was conducted in accordance with the local legislation and institutional requirements.

## Author contributions

LP: Conceptualization, Data curation, Formal analysis, Methodology, Software, Writing – original draft, Writing – review & editing, Funding acquisition, Visualization. TF: Data curation, Methodology, Writing – review & editing. ST: Conceptualization, Methodology, Project administration, Writing – review & editing. JF: Data curation, Formal analysis, Investigation, Methodology, Writing – review & editing. UA: Data curation, Formal analysis, Investigation, Software, Visualization, Writing – review & editing. AC: Funding acquisition, Resources, Supervision, Validation, Writing – review & editing. LP: Funding acquisition, Resources, Supervision, Validation, Writing – review & editing.
